# Preparation of Phosphate Glass by the Conventional and Microwave Melt-Quenching Methods and Research on Its Performance

**DOI:** 10.3390/ma18051079

**Published:** 2025-02-28

**Authors:** Baiyi Li, Guangdong Zhou

**Affiliations:** College of Chemistry, Jilin University, Changchun 130061, China; liby22@mails.jlu.edu.cn

**Keywords:** phosphate glass (PG), structure, slow release, chemical durability, scale inhibition

## Abstract

In this study, various phosphate glasses (PGs) within the CaO-Na_2_O-P_2_O_5_ and SiO_2_-CaO-Na_2_O-P_2_O_5_ systems were synthesized using both conventional and microwave melt-quenching techniques. The physical, thermal, and structural properties of these phosphate glasses, as well as their dissolution behaviors under varying temperatures and solvent conditions, were thoroughly examined. Additionally, the influence of scale inhibitor dosage, temperature, and pH on the rate of scale inhibition was assessed using a static scale inhibition test. The morphology and crystal types of the precipitates were characterized using Scanning Electron Microscopy (SEM) and X-ray Diffraction (XRD) analysis. The findings indicate that the PG structure predominantly consists of Q^2^ structural units, with a minor presence of Q^1^ units. The dissolution rate of PG escalates with an increase in temperature and a decrease in pH. Conversely, the scale inhibition efficiency diminishes with rising temperature and pH. Optimal scale inhibition efficiency, reaching up to 95.4%, was observed at a pH of 7 and a temperature of 50 °C. Under the influence of the PG scale inhibitor, the primary crystal form of CaCO_3_ was altered from calcite to vaterite.

## 1. Introduction

Phosphate glass (PG) has been extensively researched due to its low melting and glass transition temperatures, minimal light scattering, high refractive index, and substantial thermal expansion coefficient. These properties offer broad application prospects in both fundamental scientific research and industrial production [1,2,3,4,5,6,7,8]. In the field of biomedicine, bioactive PG is renowned for its excellent biocompatibility and bioabsorbability [9,10], exhibiting significant potential for applications in soft tissue engineering, wound healing, and angiogenesis promotion [11,12,13,14,15]. A key advantage of PG lies in its aqueous solubility and the ability to tailor its dissolution rate by modifying the glass composition. This customization allows the material’s degradation lifetime to be synchronized with the tissue repair process [16,17]. Phosphate glasses are increasingly utilized in hard tissue engineering, controlled release systems, and scale inhibition materials [18], and can also be integrated with polymers to create fully degradable polymer–glass composites [19,20,21].

In recent years, the formation of scale on heat transfer surfaces has emerged as a significant challenge, commonly occurring in various industrial operations, including power generation, water transport, and oil or gas production [22,23,24]. Scale formation reduces heat transfer efficiency, escalates energy consumption [25], and can lead to unexpected equipment downtime. The predominant and most effective approach for preventing scale is the application of scale inhibitors.

Phosphate glass has gained attention in the realm of water treatment due to its distinctive properties. When compared to traditional commercial-scale inhibition materials, such as organic phosphonates and copolymers, phosphate glass presents several advantages. It offers a slow-release mechanism that enables the sustained release of scale-inhibiting active components, providing prolonged scale inhibition effects, whereas traditional scale inhibitors necessitate frequent reapplication. Moreover, phosphate glass is compatible with a wide range of pH levels and high-temperature conditions, unlike some organic scale inhibitors that may degrade or fail under harsh conditions. Phosphate glass is also environmentally benign, composed of non-toxic and biodegradable elements, thereby mitigating the risk of secondary pollution often associated with organic scale inhibitors. Furthermore, the preparation process of phosphate glass is straightforward and cost-effective, enhancing its economic viability and scalability. Consequently, phosphate glass represents a promising new agent for water treatment. The chemical durability of phosphate glasses, however, is often considered to be compromised due to the asymmetry of its fundamental building block [26]. With phosphorus in a +5 valence state, the PO_4_ tetrahedron features a P=O bond that disrupts one of the upper corners of the tetrahedron. The structural classification of the PO_4_ tetrahedron may be delineated based on the number of bridging oxygens (BO) in its unit [27]. As the oxygen to phosphorus ratio (O/P) increases, indicating more bonds between neighboring phosphate tetrahedra, the number of bridging oxygens decreases, and the Q^i^ species transition in the sequence Q^3^→Q^2^→Q^1^→Q^0^, where “i” denotes the number of BO [28,29,30,31].

PG is conventionally synthesized using the traditional melt-quenching technique, which is characterized by lengthy melting durations and high energy consumption. In recent years, the application of microwave heating in the sintering of ceramics and metals has garnered increasing attention due to its rapid volumetric heating, enhanced productivity, and reduced energy usage [32,33,34]. Comparative studies between conventional melting methods and microwave melting techniques for the synthesis of PG are scant. Thus, this study employs both conventional melting and microwave melting methods to synthesize PG. Characterization of PG was conducted using infrared spectroscopy and Raman spectroscopy, allowing for an investigation into how different compositions influence the structure of the glass and its subsequent properties. The dissolution behaviors and scale inhibition properties of PG under various temperatures and pH levels were examined to assess its controlled release characteristics and the impact of temperature and pH on its release behavior and scale inhibition performance. Based on experimental observations, a mechanistic hypothesis regarding the controlled release and scale inhibition mechanisms of PG is proposed.

## 2. Materials and Methods

### 2.1. Materials

The following reagents were used: ammonium dihydrogen phosphate (NH_4_H_2_PO_4_), sodium hexametaphosphate ((NaPO_3_)_6_), calcium oxide (CaO), silicon dioxide (SiO_2_), sodium carbonate (Na_2_CO_3_), and distilled water. All reagents were sourced from Shanghai Aladdin Biochemical Technology Co., Ltd., Shanghai, China, and were of analytical grade. They were used as received without further purification.

### 2.2. Preparation of PG

Phosphate glass samples were synthesized in two different glass-forming systems: the PC system and the PS system. The PC system is defined by the formula xCaO-(50-x)Na_2_O-50P_2_O_5_ and the PS system by ySiO_2_-20CaO-30Na_2_O-(50-y)P_2_O_5_, where x ranges from 5 to 20 mol% and y from 1.5 to 6 mol%. The synthesis was carried out using conventional melt-quenching and microwave melt-quenching methods. (NaPO_3_)_6_ and NH_4_H_2_PO_4_ served as the primary raw materials, while CaO, SiO_2_, and Na_2_CO_3_ were used as auxiliary materials. A specified amount of these raw materials was weighed, thoroughly mixed, and ground in a mortar until homogeneous. The mixture was then divided into two equal portions. Each portion was placed in a quartz crucible and dried slowly at 300 °C for 30 min to remove volatile components such as H_2_O and NH_3_, which helps prevent excessive foaming during the melting process. Following pre-firing, one portion was melted in a muffle furnace at 1200 °C for 1.5 h. After melting, the molten glass was poured into a stainless steel mold to take shape. The other portion was quickly melted in a microwave oven at high power (700 W, 2.45 GHz) for 20 min, and similarly cast in a stainless steel mold. The formed samples were then placed in a desiccator for storage. Table 1 provides the sample numbers and chemical compositions for the two PG systems.

### 2.3. Characterization of Materials

The crystalline phases of the PG sample and the CaCO_3_ scale were analyzed using an Empyrean powder X-ray diffractometer (PANalytical B.V., Almelo, The Netherlands) equipped with a CuKα radiation source (λ = 1.5418 Å). The operational settings were a voltage of 40 kV and a current of 30 mA, with a scan range of 2θ from 10° to 80° and a scanning speed of 4°/min.

Fourier-transform infrared spectroscopy (FTIR) was performed on the samples using a Nicolet IS5 spectrometer (Thermo Scientific, Waltham, MA, USA). The samples were finely ground, mixed with potassium bromide (KBr) in a ratio of 1:100, and then compressed into translucent pellets. Each sample underwent 32 scans within a wavelength range of 4000 to 400 cm^−1^, with a resolution of 0.4 cm^−1^.

Raman spectroscopy was conducted using a LabRAM HR Evolution high-resolution laser Raman spectrometer (HORIBA France SAS, Paris, France). The instrument was set with a 532 nm laser, a scanning range of 200–1400 cm^−1^, a laser power of 80 mW, a resolution of 0.6 cm^−1^, and an acquisition time of 60 s.

Differential thermal analysis (DTA) was carried out on a NETZSCH STA 2500 analyzer (NETZSCH Geratebau GmbH, Selb, Germany). The PG sample, weighing 5 mg, was heated from 25 °C to 800 °C at a rate of 10 °C/min under an airflow of 50 mL/min.

The density of the samples was measured using the Archimedes principle, with anhydrous ethanol as the immersion medium.

The morphological characteristics of the CaCO_3_ scale were investigated using a Quattro E SEM scanning electron microscope (Thermo Scientific, Waltham, MA, USA). Prior to imaging, the samples were sputter-coated with gold to enhance conductivity. Imaging was conducted at an accelerating voltage of 3 kV, a beam current of 50 pA, and a working distance of 10 mm, using secondary electron detection mode at a magnification of 2000×.

### 2.4. Dissolution Test

#### 2.4.1. Determination of the Slow-Release Dissolution Rate of PG

The slow-release dissolution rate of PG was determined using a gravimetric method. The scale inhibitor samples were polished, dried in an oven, and then submerged in a constant temperature water bath at 50 °C. Periodically, a specified volume of the water was sampled for analysis of total phosphorus and orthophosphorus concentrations. After each sampling, an equivalent volume of deionized water was replenished. Following a predetermined duration (*t* days), the samples were removed, dried for 2 h in an oven, and weighed. The dissolution rate (*r*) of PG was calculated using the equation:

(1) $$r=\frac{W_{1}-W_{2}}{S\times t}$$

where *r* denotes the dissolution rate of the sample (mg∙cm^−2^∙d^−1^); *S* represents the surface area of the sample (cm^2^); *t* indicates the dissolution time of the sample (d); *W*_1_ and *W*_2_ are the masses of the sample before and after dissolution (mg), respectively. Each dissolution rate determination was replicated three times to minimize measurement errors.

#### 2.4.2. Effect of Temperature and pH on Dissolution Rate

To explore the impact of temperature on the dissolution behavior of phosphate glass samples, the reaction temperatures were maintained at 30 °C, 50 °C, 60 °C, and 70 °C using a constant-temperature water bath. Additionally, the influence of various pH values (4, 6, 7, 9) on the dissolution rate was examined at 50 °C. Detailed methodologies for these measurements are provided in Section 2.4.1.

#### 2.4.3. Measurement of Orthophosphate and Total Phosphorus

The digestion of total phosphorus in the water samples was facilitated using potassium persulfate, followed by quantification via the ammonium molybdate spectrophotometric method. Orthophosphate levels were directly measured using the same method. The calculation for polyphosphate (expressed as PO_4_^3−^) involved subtracting the orthophosphate content from the total phosphate content. All chemical reagents employed were prepared according to the protocol outlined in the national standard “Determination of Total Phosphorus in Water Quality in China” (GB11893-89). Measurements were conducted at a wavelength of 710 nm using a 1 cm cuvette.

### 2.5. Test of Scale Inhibition

#### 2.5.1. Measurement of the Scale Inhibition Efficiency of PG

According to GB/T 16632-2019, the static scale inhibition efficiency of the sample was evaluated using the calcium carbonate deposition method. A 500 mL volumetric flask was filled with standard solutions of CaCl_2_ and NaHCO_3_, followed by the addition of the water treatment agent test solution and 20 mL of borax buffer. A blank control was prepared under identical conditions, excluding the water treatment agent. The mixture was then maintained in a constant-temperature water bath for 10 h. The CaCO_3_ scale was collected using filter paper and dried for further analysis. The remaining Ca^2+^ concentration was titrated with an EDTA solution. The scale inhibition rate of PG was calculated using the following formula:

(2) $$Q=\frac{C_{2}-C_{1}}{C_{0}-C_{1}}$$

where *C*_2_ denotes the final remaining Ca^2+^ concentration after adding the PG scale inhibitor, expressed in mg/mL, *C*_1_ is the final Ca^2+^ concentration in the blank control group, expressed in mg/mL, and *C*_0_ is the initial Ca^2+^ concentration in the solution, expressed in mg/mL.

#### 2.5.2. Effect of Temperature and pH on Scale Inhibition Efficiency

Following preliminary tests, the optimal concentration of the scale inhibitor was selected for further evaluation of its performance. The reaction temperatures of 30 °C, 50 °C, 60 °C, and 70 °C were controlled using a constant-temperature water bath to assess the effects of temperature on the scale inhibition efficiency of phosphate glass samples. Additionally, the impact of different pH values (4, 6, 7, 9) on scale inhibition efficiency was analyzed at 50 °C. Specific measurement methods are detailed in Section 2.5.1.

## 3. Results and Discussion

### 3.1. XRD Characterization

**Figure 1:**
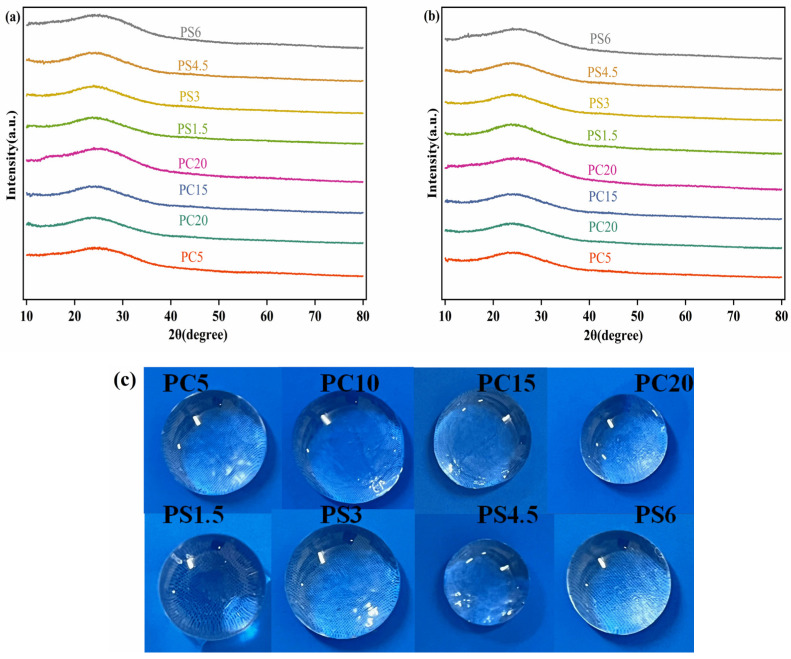
(**a**) XRD pattern of the sample prepared by conventional melt-quenching, (**b**) XRD pattern of the sample prepared by microwave melt-quenching, (**c**) representative picture of some of the obtained glasses.

Two distinct types of phosphate glasses, designated as PC and PS, were synthesized using both conventional melt-quenching and microwave melt-quenching techniques. We investigated their structural properties, slow-release efficiencies, and their capabilities in inhibiting the crystallization and scaling of CaCO_3_. Under the conditions used for synthesis, all samples resulted in homogeneous glasses. Figure 1 displays the XRD patterns of the samples prepared. The patterns are characterized by broad and diffuse peaks, with an absence of sharp peaks, confirming the amorphous and non-crystalline nature of the materials. Macroscopic photographs of the glass samples are shown in Figure 1c, with all glasses appearing colorless, visually clear, and homogeneous.

### 3.2. Structural Characteristics

#### 3.2.1. FTIR Analysis

**Figure 2:**
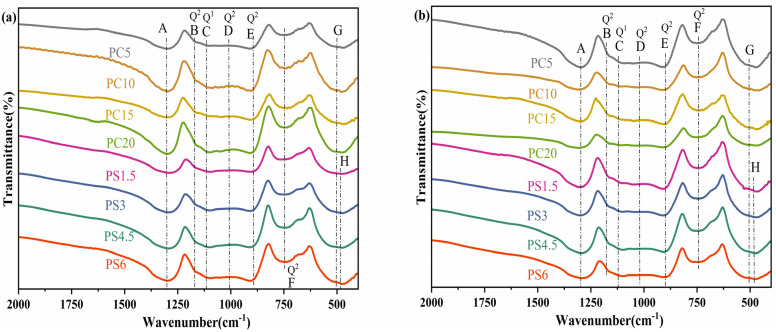
(**a**) FTIR spectrum of the sample prepared by conventional melt-quenching, (**b**) FTIR spectrum of the sample prepared by microwave melt-quenching.

Figure 2 presents the FTIR spectrum of the glass. The spectrum is dominated by a broad band and a shoulder, indicative of the lack of long-range periodicity typical of an amorphous structure. Table 2 enumerates the attributions of each absorption peak. The examination of Figure 2 reveals that the FTIR spectra of the phosphate glass samples synthesized by the two methods bear a resemblance, suggesting similar structural compositions. The characteristic peaks in the FTIR spectrum of phosphate glass include, in sequence from left to right: the absorption band at 1290 cm^−1^ (A), representing the stretching vibration of the P=O bond; the band near 1170 cm^−1^ (B), attributed to the symmetric stretching vibration of PO_2_ in the Q^2^ unit [31]. The shoulder bands at 1120 cm^−1^ (C) and 1030 cm^−1^ (D) correspond to the symmetric stretching vibration of the Q^1^ unit and the asymmetric stretching vibration of the P-O-P bond in Q^2^ unit; the shoulder near 915 cm^−1^ (E) is associated with the asymmetric stretching vibration of the P-O-P bond in the Q^2^ unit; the band near 750 cm^−1^ (F) is for the symmetric stretching of the P-O-P in the Q^2^ unit; and a bending vibration peak of the PO_4_ tetrahedron occurs at 515 cm^−1^ (G). The introduction of SiO_2_ in the PS system results in the emergence of new peaks near 499 cm^−1^ (H), which are attributed to the bending vibrations of Si-O-Si or Si-O-P bonds [35]. Four major absorption peaks at 1290 cm^−1^, 1170 cm^−1^, 915 cm^−1^, and 750 cm^−1^ are linked to the Q^2^ unit, suggesting that this unit forms the predominant structural framework of the glass. Additionally, a minor presence of Q^1^ structural units is also detected. A faint characteristic band around 1630 cm^−1^ in some samples might be due to the absorption of a small amount of moisture from the air during sample preparation [36]. In the analysis of PC system samples, an increase in the CaO content correlates with a shift in the P=O absorption peak at 1290 cm^−1^ and the P-O-P bond absorption peaks at 915 cm^−1^ and 750 cm^−1^ toward higher frequencies and shorter wavelengths. These shifts suggest a continuous strengthening of the bond strength within these functional groups, implying enhanced network connectivity and the fortification of the P-O-P bond interactions. The Ca^2+^ ion, acting as a network modifier, preferentially binds with non-bridging oxygen (NBO) to form Ca-O-P bonds. The high charge density of Ca^2+^ facilitates its simultaneous bonding with NBOs on adjacent chains, thereby bridging adjacent P-O-P chains and enhancing the degree of three-dimensional network cross-linking. This cross-linking contributes to a reduction in the number of NBOs, thereby indirectly bolstering the stability of P-O-P bonds. Additionally, the presence of CaO enhances the chemical stability of phosphate glasses; the wavenumber of the bending vibration of the PO_4_ tetrahedron at 515 cm^−1^ shifts to lower frequencies, indicative of an increase in the covalent bond character of the metal ion’s interaction with the non-bridging oxygen on the PO_4_ tetrahedron. This shift is attributable to the strong polarization capability of Ca^2+^, thereby reducing the ionic bond component of the Ca-O bond relative to that of the Na-O bond. For the PS system sample, it can be seen from the figure that the P=O stretching vibration peak, with a wavenumber of about 1290 cm^−1^, and the asymmetric stretching vibration absorption of P-O-P, with a wavenumber of about 915 cm^−1^, move to lower frequencies and longer wavelengths with increasing substitution of SiO_2_. This behavior is primarily due to the relative reduction in P_2_O_5_ content, which diminishes the P=O bond strength and disrupts the phosphorus–oxygen framework in the phosphate glass network. The incorporation of the silicate phase results in network inhomogeneity, diversification of vibration modes, and peak broadening. The symmetric stretching vibration peak of P-O-P near 750 cm^−1^ shifts toward a higher wavenumber, indicating that the formation of Si-O-P bonds increases the rigidity of the phosphate network, leading to a shortening of P-O-P bond lengths and an enhancement of bond strength. Conversely, the shift in the PO_2_ absorption peak toward higher frequencies and shorter wavelengths implies a strengthening of the bond between non-bridging oxygen and phosphorus, while the bond between non-bridging oxygen and monovalent and divalent ions weakens. Consequently, these ions are more prone to ion exchange with H^+^, resulting in diminished chemical stability of the PG and an enhanced slow-release capability in aqueous environments.

#### 3.2.2. Raman Spectroscopy Analysis

**Figure 3:**
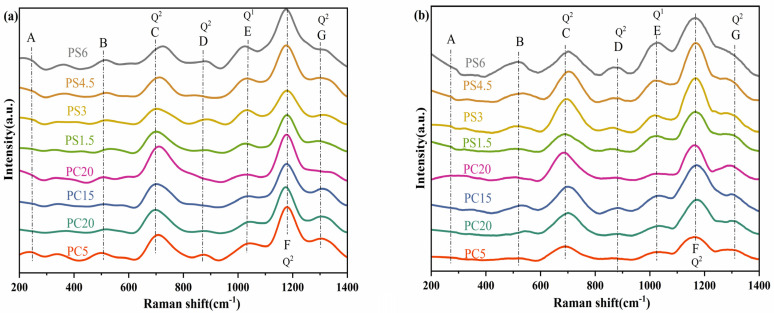
(**a**) Raman spectrum of the sample prepared by conventional melt-quenching, (**b**) Raman spectrum of the sample prepared by microwave melt-quenching.

Figure 3 illustrates the Raman spectrum of PG, characterized predominantly by vibrations associated with phosphate species. The spectrum displays various network-forming units as detailed in Table 3. Specifically, in Figure 3, the peak at approximately 270 cm^−1^ (A) is attributed to the bending vibration of the PO_4_ tetrahedron. The peak near 515 cm^−1^ (B) results from the bending vibration of P=O, while the peak at about 690 cm^−1^ (C) corresponds to the symmetric stretching vibration of P-O-P in the Q^2^ unit. Additionally, the asymmetric stretching vibration peak of the Q^2^ unit is observed near 880 cm^−1^ (D), and a weak absorption band near 1040 cm^−1^ (E) is linked to the symmetrical stretching vibration of P-O-P in the Q^1^ unit. With the incorporation of SiO_2_ into the glass composition of the PS glass system, new bands emerge near 414 cm^−1^ and 825 cm^−1^, associated with the stretching vibration of the Si-O-Si bond [38]. Although the SiO_2_ content is minimal, its influence is discernible in the spectrum. In all examined samples, the absorption peak near 1160 cm^−1^ (F) exhibits the strongest signal and is identified as the symmetric stretching vibration mode of PO_2_ in the Q^2^ unit. Additional, weaker features at higher wavelengths (G) are indicative of the asymmetric stretching vibration of PO_2_ in the Q^2^ unit [39]. The Raman spectrum of this glass aligns with the typical spectrum observed for metaphosphate glasses with a Q^2^ composition [40], confirming that the primary network structure of the glass is the metaphosphate (Q^2^) structure. This finding is consistent with infrared spectroscopy results. As the concentrations of CaO and SiO_2_ increase, the overall Raman spectrum remains predominantly characterized by Q^2^ structural units, indicating stability in the glass network structure. In the PC series samples, the marked increase in peak intensity near 690 cm^−1^, accompanied by a shift to higher wavenumbers, can be attributed to the bridging effect of Ca^2+^ ions. These ions enhance the cross-linking of phosphate chains, thereby elevating the symmetric vibrational frequency of P-O-P bonds. For the PS series samples, the observed blue shift in the P-O-P symmetric stretching vibration peak with increasing SiO_2_ content suggests enhanced bond strength within P-O-P linkages. Concurrently, the progressive intensification of the 1040 cm^−1^ peak implies an increasing proportion of Q^1^ units in the glass network, indicating a structural evolution. This evolution occurs as SiO_2_ acts as a network former, partially replacing P_2_O_5_ and thus increasing the oxygen-to-phosphorus ratio (O/P). The higher O/P ratio facilitates a Q^2^→Q^1^ structural transition through the depolymerization of phosphate chains.

### 3.3. Physical and Thermal Properties

The density of glass is determined by the mass of its constituent atoms and is influenced by factors such as the coordination number and the degree of atomic packing. The densities of two series of glass samples, prepared during our experiment, are presented in Table 4. Both series of samples demonstrate a consistent pattern: as the concentration of added extra-network CaO increases, the density of the PC series samples also increases. Calcium (Ca^2+^) acts as an extra-network ion with a coordination number of six. It does not contribute to network formation but fills the voids within the network. Ca^2+^ exhibits low reactivity within the network structure, but it has a cumulative effect that increases cross-linking within the glass structure, thereby raising the density as the CaO content increases. For the PS system glass, when the addition of SiO_2_ is less than 4.5 mol%, there is a slight increase in glass density with the amount of SiO_2_ added, peaking at 2.548 g/cm^3^. However, when the addition reaches 6 mol%, the density decreases. This decrease in density occurs because, at low addition levels, SiO_2_ forms [SiO_4_] tetrahedrons, which enter the network to strengthen the network connection, making the internal structure tend to be denser. Conversely, with further increases in SiO_2_, the structure of the glass alters; the [SiO_4_] tetrahedron-formed silicate network becomes independent, isolating and fragmenting the phosphate network, thus reducing the overall compactness. The density of the PG samples prepared by both conventional melt-quenching and microwave melt-quenching methods decreases with the addition of SiO_2_. In addition, the reference shows that [42] the porosity of the glass is also closely related to the density, and the final glass porosity depends largely on the average density of the sample. At lower density, the size of the pore structure is larger, and most of the pores are interconnected. However, except for samples PC5 and PS1.5, the densities of samples prepared by the microwave method are slightly higher than those prepared by the conventional method. This discrepancy can be attributed to the microwave melting method, a non-contact technology in which heat is transferred via electromagnetic waves. This method generates a significant amount of heat during the sintering process, which is transferred to the material’s interior, accelerating the densification process and achieving similar or higher densities in a shorter time [43].

**Figure 4:**
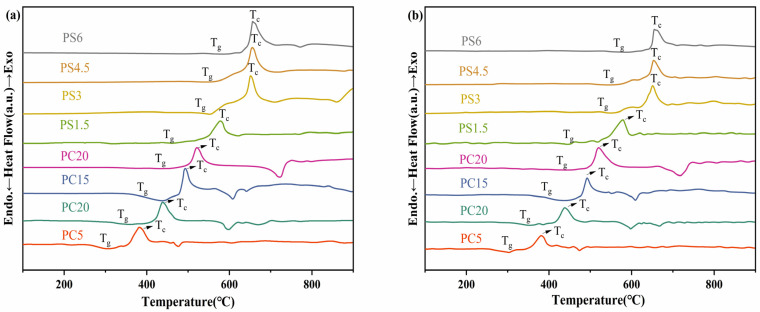
(**a**) DTA curve of the sample prepared by conventional melt-quenching, (**b**) DTA curve of the sample prepared by microwave melt-quenching.

The DTA curves and thermal analysis results for the PG samples are presented in Figure 4 and Table 4. Due to their similar structures, the glass transition temperature (T_g_) and crystallization temperature (T_c_) values of the PG samples prepared by the two methods are comparable. The T_g_ of the PC system glass samples varies with the degree of CaO substitution. For example, in PC5, the T_g_ is approximately 280 °C, and the T_c_ is about 380 °C. The incorporation of CaO increases both T_g_ and T_c_, an effect that is more pronounced in the PC10 and PC15 samples. Ca^2+^ serves as an ionic cross-linking agent among non-hybridized oxygens in two distinct chains. Adding CaO to the composition results in the formation of stronger P-O-Ca bonds (bond dissociation energy: ~320–380 kJ/mol for P-O-P vs. ~420–460 kJ/mol for P-O-Ca), which, in turn, enhances the T_g_ by increasing the cross-linking density and thereby stiffening the glass network. SiO_2_ acts as a network former in glass. In the PS systems, the addition of SiO_2_ leads to the formation of [SiO_4_] tetrahedra within the structure, which establishes stronger bonds and leads to increased T_g_ and T_c_. At low SiO_2_ contents, Si^4+^ ions are evenly dispersed within the phosphate network, effectively eliminating phase-boundary defects and maintaining structural homogeneity. This uniformity contributes to a concurrent enhancement of both T_g_ and T_c_. When the SiO_2_ content exceeds 4.5 mol%, thermodynamic immiscibility between SiO_2_ and P_2_O_5_ induces phase separation. The high rigidity of silicate-rich microdomains further raises T_g_, while the growth rate of T_c_ is reduced due to restricted crystallization kinetics in phase-separated systems.

### 3.4. Dissolution Behavior

Hydration and hydrolysis reactions are crucial in the dissolution process of PG in a liquid medium [26]. The hydration reaction involves an ion exchange between mobile ions, such as Na^+^ in the glass and H^+^ in the solution, ultimately forming a hydration layer at the glass-solution interface. Under the influence of H^+^ and H_2_O, the P-O-P bond in the hydration layer breaks, leading to hydrolysis and the formation of phosphate chains of varying lengths that dissolve in water. This process is accelerated by an increase in H^+^ concentration, making the dissolution behavior of PG heavily dependent on the ambient pH. This study thus explores the dissolution characteristics with respect to weight loss, pH, temperature, and phosphorus release.

#### 3.4.1. Weight Loss

**Figure 5:**
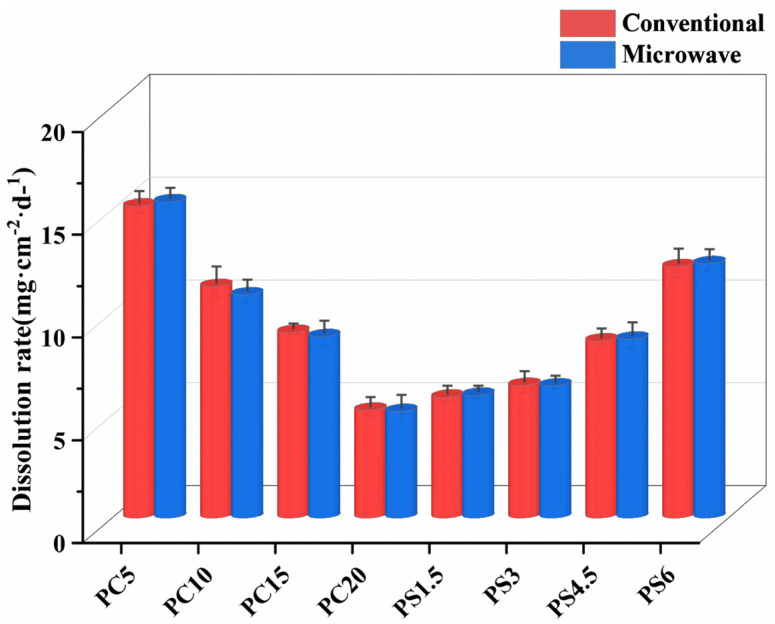
Dissolution rate of PG.

The weight loss of the glass surface is a crucial parameter for assessing its chemical stability. In the context of slow-release phosphate glass, this loss represents the macroscopic rate at which beneficial components are released. This rate is primarily quantified by measuring the weight loss per unit area over time. The results from tests on the dissolution rate of PC and PS samples in 50 °C deionized water are illustrated in Figure 5. Both samples, prepared using conventional and microwave melt-quenching methods, displayed similar dissolution rates. In the PC glass system, the PC5 sample demonstrated the fastest dissolution rate. Conversely, the PC20 sample, with a higher CaO content, showed the slowest release rate. Despite the fact that adding CaO increases the number of non-bridging oxygen atoms in the glass structure—potentially disrupting its layered or closed-chain structure—it does not diminish the structural stability. This is because the Ca^2+^ ions fill the network gaps, compacting the structure and effectively reducing the diffusion rate of water molecules within these gaps. This action slows the formation and progression of the glass hydration layer. Additionally, due to the chelating effect of phosphate, Ca^2+^ exhibits a much stronger field intensity compared to Na^+^, enabling it to create non-bridging oxygen links between adjacent [PO_4_] tetrahedra. This not only strengthens the network connections but also enhances the strength of the P-O-P bonds, thereby fortifying the overall network structure of the sample. For the PS system, the addition of SiO_2_ results in an increased dissolution rate. When the substitution amount is below 4.5 mol%, the increase in dissolution rate is relatively gradual. However, at a substitution level of 6 mol%, the rate of release notably accelerates. This phenomenon can be attributed to the fact that, with a small amount of SiO_2_ substitution, [SiO_4_] integrates into the phosphate network, forming a P-O-Si bond that is susceptible to hydrolysis [44]. Nonetheless, as the SiO_2_ content rises, Si^4+^ and Na^+^ separate from the metaphosphate chain network, loosening the internal connections within the glass structure and significantly reducing its chemical stability.

#### 3.4.2. Effect of pH and Temperature on Dissolution Rate

**Figure 6:**
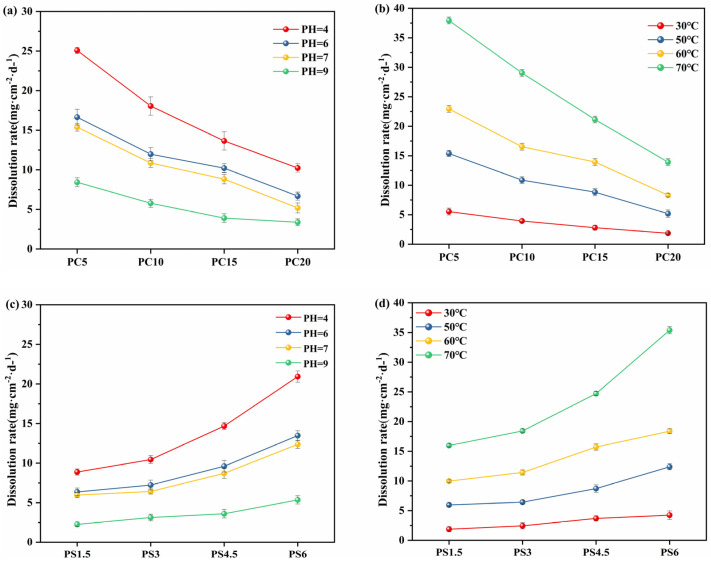
(**a**,**c**) Effect of pH on dissolution rate, (**b**,**d**) Effect of temperature on dissolution rate.

Figure 6 illustrates the impact of pH and temperature on the slow-release performance of PG. The data indicate that the dissolution rate of PG increases significantly with rising temperature. This increase can be attributed to enhanced ion exchange, where Na^+^ from the tetrahedral [PO_4_] units are replaced by H^+^ in the water, thereby accelerating the slow-release rate. Conversely, the dissolution rate decreases as the pH increases. The dissolution mechanism begins and progresses through the interaction of water (H_2_O) and protons (H^+^ or H_3_O^+^) at the interface between the glass and the solvent. Protons and water molecules collaboratively attack the P-O-P bonds within the hydration layer, leading to the breakdown of the glass network structure and the formation of phosphate chains with varying degrees of polymerization. These chains then dissolve into the water, a process that is macroscopically observed as the dissolution of the glass. In acidic environments, the presence of protons facilitates the dissociation of P-O-M bonds (where M represents metal ions) more efficiently than in neutral conditions. This results in a faster hydration and dissolution process [45]. Therefore, lowering the pH enhances the dissolution rate. Furthermore, experimental observations revealed that the pH of the solution decreased during the dissolution of phosphate glass, supporting the theory that dissolution occurs through the disruption of the P-O-P chain within the network structure.

#### 3.4.3. Phosphorus Release

**Figure 7:**
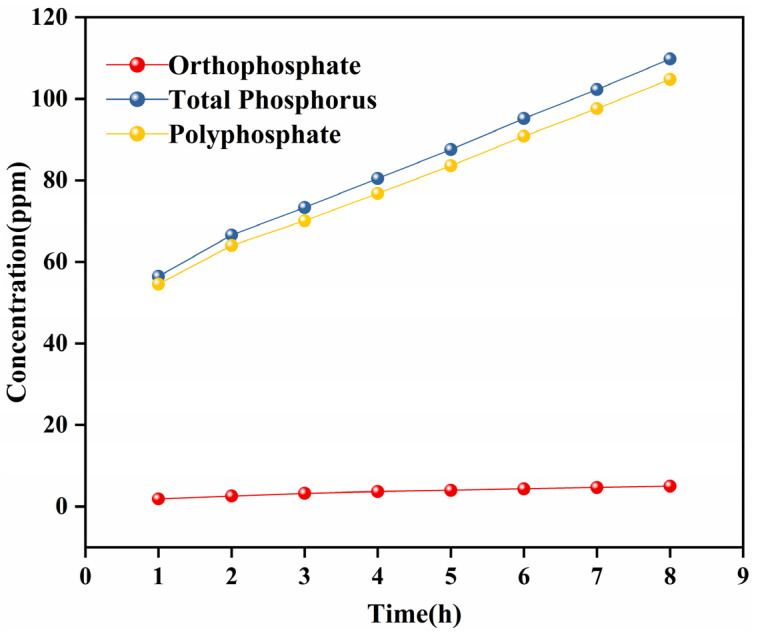
Relationship between total phosphorus, orthophosphate, and polyphosphate concentrations over time.

Figure 7 illustrates the concentrations of total phosphorus, orthophosphate, and polyphosphate in the dissolved water samples from the PC5 sample at 50 °C. Since the release patterns of all samples are similar, the discussion will focus solely on the PC5 sample. Throughout the dissolution process, the concentrations of various phosphorus ions consistently increased over time. The non-zero intercepts of the straight lines in Figure 7 suggest that the water treatment agent has a relatively high initial dissolution rate and exhibits parabolic time dependence. After a brief period (within one hour), a steady state is achieved, followed by a constant, linear release process. Oosterbeek et al. [46] categorized the dissolution process into two kinetic stages based on the mass loss curve: the initial stage, referred to as the t^1/2^ stage, is followed by a stage where dissolution is linearly related to time. Once the phosphate chains are completely surrounded by water, they detach from the partially hydrated chains and dissolve, leading to an increased dissolution rate at the start of the process. As the dissolution progresses, the hydration layer stabilizes, allowing the dissolution to continue linearly with time. This characteristic of a constant dissolution rate is particularly beneficial in practical applications, as it enhances the controllability of PG slow release. This controlled release mechanism can gradually and consistently release polyphosphate, maintaining an effective concentration level over an extended period. By using time as a parameter, the quantity of effective ions in the solution at different intervals can be calculated, facilitating precise control over the timing and dosage of additions.

### 3.5. Evaluation of Scale Inhibition Performance

**Figure 8:**
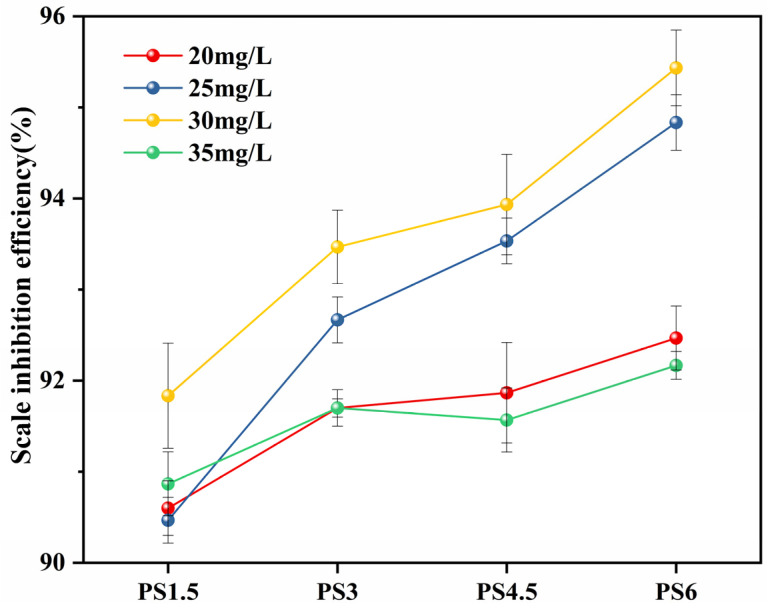
Effect of sample concentration on scale inhibition efficiency at 50 °C.

Figure 8 illustrates the scale inhibition performance of the PS series samples at varying additive concentrations. It is observed that the scale inhibition efficiency peaks at 95.3% when the additive concentration of the PS6 sample is 30 mg/L. However, increasing this concentration to 35 mg/L results in a reduced efficiency of 92.2%. This reduction can be attributed to the threshold effect, suggesting that the dosage has surpassed the optimal concentration, beyond which further increases do not enhance the scale inhibition efficiency. Similarly, the maximum scale inhibition efficiency for the other three samples is also achieved at an additive concentration of 30 mg/L. Consequently, we have identified 30 mg/L as the optimal concentration for scale inhibition in subsequent experiments.

#### 3.5.1. Scale Inhibition Efficiency at Optimal Scale Inhibition Concentration

**Figure 9:**
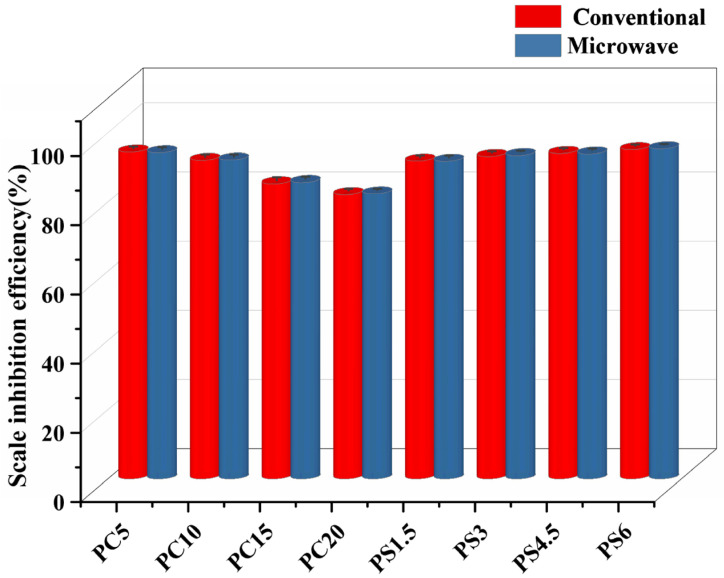
Scale inhibition efficiency of PG.

Scale inhibition experiments were conducted using PC and PS series samples at 50 °C with a concentration of 30 mg/L. The results, as depicted in Figure 9, show that the scale inhibition efficiencies of PG samples prepared by both conventional melt-quenching and microwave melt-quenching techniques are comparable. For the PC series samples, the scale inhibition performance deteriorates significantly when the CaO content exceeds 15 mol%. This decline is due to the dissolution of the scale inhibitor in the solution, which simultaneously releases Ca^2+^ from the sample. As a scale-forming ion, an increase in CaO content raises the Ca^2+^ concentration in the solution, hastening the supersaturation of scale-forming ions and subsequent crystallization. The PS series samples demonstrate a continual improvement in scale inhibition with an increase in SiO_2_ content. During scale formation, [SiO_4_] tetrahedra from phosphate glass integrate into the CaCO_3_ crystal lattice. The structural and dimensional mismatch between the [SiO_4_] tetrahedra and the native CaCO_3_ lattice units causes lattice distortion, which disrupts the regular crystal growth pattern. This distortion results in thermodynamic instability, effectively inhibiting the formation of large, densely packed scale crystals and thereby enhancing scale inhibition efficiency. Additionally, SiO_2_ in aqueous solutions forms nanoscale colloidal particles or exhibits surface-active properties. These colloidal or amphiphilic entities adsorb onto scale-forming ions or nascent CaCO_3_ nuclei via specific surface interactions, inducing electrostatic repulsion between particles. Such repulsion kinetically impedes the collision-coalescence processes, preventing the agglomeration of micron-scale crystalline particulates.

#### 3.5.2. Effect of pH and Temperature on Scale Inhibition

**Figure 10:**
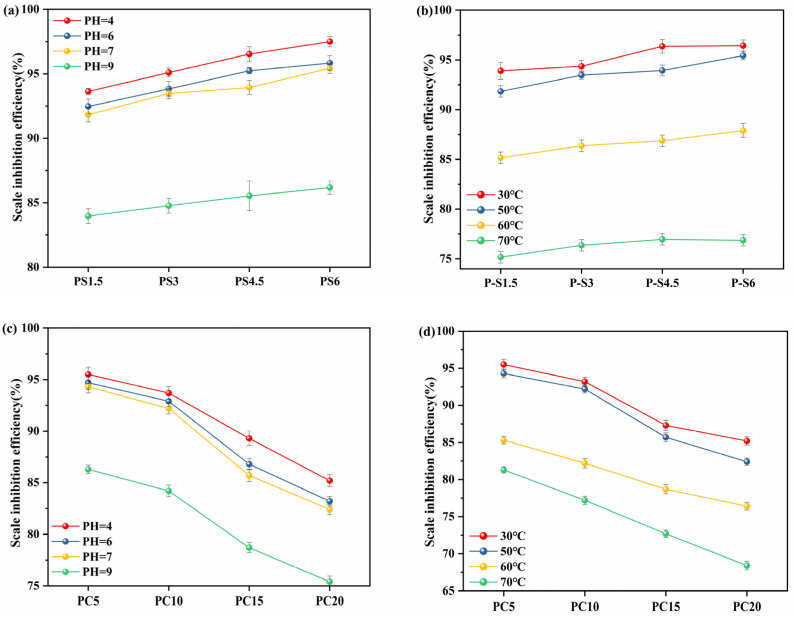
(**a**,**c**) Effect of pH on scale inhibition efficiency, (**b**,**d**) Effect of temperature on scale inhibition efficiency.

To examine the impact of temperature on scale inhibition performance, we maintained consistent conditions across all tests involving PG samples. Figure 10b,d illustrate that the scale inhibition efficiency is significantly influenced by the reaction temperature. As the temperature increases, the scale inhibition efficiency decreases. At 30 °C, the scale inhibition efficiency reaches its peak, with the PS6 sample achieving an efficiency of 96.4%. However, at 70 °C, the highest efficiency observed in the PS series samples drops to 76.9%, with PC20 recording as low as 68.4%. This decrease in efficiency is attributed to the well-known fact that the solubility of CaCO_3_ decreases with rising temperatures. Additionally, higher temperatures accelerate the formation of a thicker and faster-forming scale layer, further diminishing the scale inhibition efficiency. The influence of pH on scale inhibition efficiency was also investigated, maintaining a temperature of 50 °C and a sample concentration of 30 mg/L. As shown in Figure 10a,c, the scale inhibition efficiency gradually decreases as the pH value increases from 4 to 9. Under alkaline conditions, the efficiency is notably lower compared to acidic conditions. This difference is due to the high concentrations of H^+^ ions under acidic conditions, which interact with CO_3_^2−^, preventing it from combining with Ca^2+^ to form insoluble calcium carbonate.

#### 3.5.3. Characterization of CaCO_3_ Crystals by SEM and XRD

**Figure 11:**
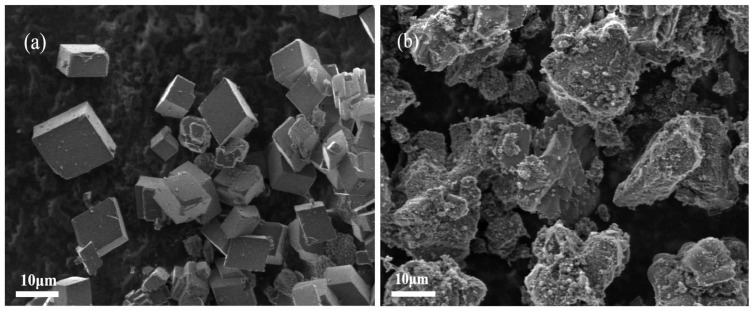
SEM micrographs of CaCO_3_ precipitation without (**a**) and with (**b**) scale inhibitor.

To investigate the mechanism of scale inhibition, we first examined the morphology of CaCO_3_ crystals using an SEM. As shown in Figure 11a, untreated CaCO_3_ crystals exhibit a regular rhombic hexahedral shape with smooth surfaces and oriented growth. However, upon treatment with the PG scale inhibitor, the morphology of these crystals altered dramatically. The crystal surfaces became rough and uneven, exhibiting numerous defects, as depicted in Figure 11b. The formation of CaCO_3_ crystals involves three stages: seed crystal formation, nucleation, and crystal growth. Inhibiting any of these stages can effectively prevent scale formation. The PG scale inhibitor dissolves in water and releases a large amount of polyphosphate. This polyphosphate can bind extensively with Ca^2+^ ions in the solution, significantly reducing the formation of CaCO_3_ crystals. Additionally, polyphosphates can adhere to the exposed Ca^2+^ on the surfaces of CaCO_3_ nuclei, forming a barrier layer that impedes the growth of existing CaCO_3_ crystals and disrupts their orientation. This interference with normal crystal lattice growth helps achieve effective scale inhibition.

**Figure 12:**
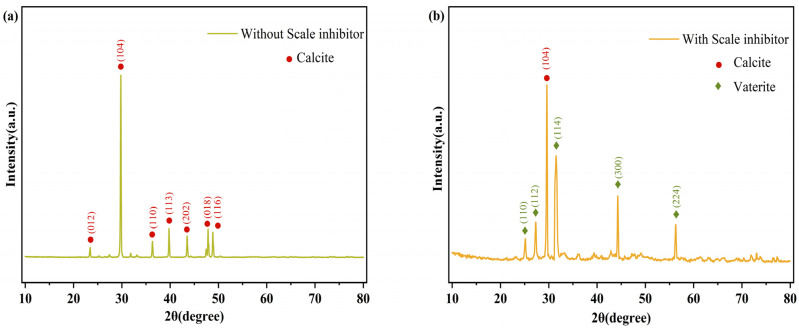
XRD patterns of CaCO_3_ precipitation without (**a**) and with (**b**) scale inhibitor.

Further analysis was conducted using X-ray diffraction (XRD). The XRD pattern shown in Figure 12a indicates that, without the scale inhibitor, the peaks at (012), (104), (110), (113), (202), (018), and (116) correspond to the characteristic planes of calcite, the most thermodynamically stable form of CaCO_3_. Following the addition of the PG scale inhibitor, new characteristic crystal planes at (110), (112), (114), (300), and (224) emerge in Figure 12b, which are indicative of vaterite. Compared to calcite, vaterite features a more loosely packed structure with a higher solubility product and greater free energy, making it easier to dissolve and wash away with water. This transformation suggests that the PG scale inhibitor causes lattice distortion in CaCO_3_, corroborating the SEM findings.

## 4. Conclusions

In this study, two different PG systems were synthesized using the conventional melt-quenching method and the microwave melt-quenching method. We investigated the physical, thermal, and structural properties of the glasses in both systems, as well as their dissolution behavior at varying pH levels and temperatures, and their capability to inhibit the crystallization of CaCO_3_ into scale. Additionally, the mechanisms behind their slow-release dissolution and scale inhibition were analyzed. We also explored the impact of compositional changes on chemical durability by correlating these changes with alterations in the glass network structure. The following conclusions were reached:

(1) PG prepared by both methods exhibits similar structural and thermal properties, as well as comparable slow-release dissolution behavior and scale inhibition performance. However, due to microwaves accelerating the melt homogenization process and reducing structural defects, glass synthesized by the microwave-quenching method is slightly denser than that prepared by the conventional melt-quenching method. Under the condition of achieving the same density and performance, when using the microwave melting and quenching method, the melting time is shortened by approximately 78%, and the energy consumption is reduced by about 85%.
(2) Structural and thermal property data: FTIR and Raman spectroscopic analyses confirmed that PG prepared by two methods (traditional melt-quenching and microwave melt-quenching) exhibited essentially identical structural characteristics, primarily consisting of Q^2^ structural units. Density, glass transition temperature (T_g_), and crystallization temperature (T_c_) data indicated regular changes in glass density and thermal stability with increasing CaO and SiO_2_ content. Specifically, the addition of SiO_2_ enhanced the strength of P-O-P bonds, while the substitution of CaO increased glass cross-linking density and T_g_ by forming stronger P-O-Ca bonds.
(3) Dissolution behavior data: dissolution experiments conducted at various temperatures (30–70 °C) and pH (4–9) values revealed the slow-release characteristics of PG. Specifically, the decrease in pH accelerated the dissolution rate of PG, while increasing the temperature, although it accelerated the dissolution process and reduced its scale inhibition efficiency. The chemical stability of PG was quantified by measuring weight loss per unit area per unit time. Dissolution in the glass occurs through the breaking of P-O-P chains in the phosphate glass network structure. Initially, dissolution exhibits a high rate of parabolic time dependence, which quickly transitions to a linear dissolution phase.
(4) Scale inhibition performance data: static scale inhibition experiments were conducted to assess the scale inhibition efficiency of PG. The results showed that the PS glass system exhibited a scale inhibition efficiency exceeding 90% at 50 °C, with some samples achieving even higher efficiencies of over 95%. PG inhibits scale formation for two reasons: firstly, the polyphosphate produced by the hydrolysis of PG complexes with Ca^2+^ in the solution, reduces the concentration of free Ca^2+^ and prevents the crystallization of CaCO_3_ into limescale. Secondly, it induces lattice distortion in already-formed CaCO_3_, altering the crystal form from calcite to vaterite, which results in a looser structure that is more easily removed by flowing water.

## Figures and Tables

**Table 1:** Theoretical composition of phosphate glass samples from different systems.

Glass System	Sample Code	Theoretical Composition (mol%)			
		P_2_O_5_	Na_2_O	CaO	SiO_2_
PC	PC5	50	45	5	-
	PC10	50	40	10	-
	PC15	50	35	15	-
	PC20	50	30	20	-
PS	PS1.5	48.5	30	20	1.5
	PS3	47	30	20	3
	PS4.5	45.5	30	20	4.5
	PS6	44	30	20	6

**Table 2:** FTIR peaks attribution of phosphate glass samples.

Peak	Attribution
A	Asymmetric stretching vibration of P=O bond [36]
B	Symmetric stretching vibration of PO_2_ in Q^2^ unit [31]
C	Symmetric stretching vibration of Q^1^ unit [36]
D	Asymmetric stretching vibration of P-O-P bond in Q^2^ unit [31]
E	Asymmetric stretching vibration of P-O-P bond in Q^2^ unit [31]
F	Symmetric stretching vibration of P-O-P bond in Q^2^ unit [31]
G	Bending vibration of the PO_4_ tetrahedron [37]
H	Bending vibration of Si-O-Si or Si-O-P bond [35]

**Table 3:** Raman peaks attribution for phosphate glass samples.

Peak	Attribution
A	Bending vibration of the PO_4_ tetrahedron [41]
B	Bending vibration of P=O bond [31,39]
C	Symmetric stretching vibration of P-O-P bond in Q^2^ unit [39,41]
D	Asymmetric stretching vibration of P-O-P bond in Q^2^ unit [39]
E	Symmetric stretching vibration of P-O-P in Q^1^ unit [39]
F	Symmetric stretching vibration of PO_2_ in Q^2^ unit [41]
G	Asymmetric stretching vibration of PO_2_ in Q^2^ unit [39,41]

**Table 4:** Density (ρ), glass transition temperature (T_g_), crystallization temperature (T_c_) of PG.

Glass System	Sample Code	ρ(g/cm^3^) ± 0.001		T_g_ (°C) ± 2		T_c_ (°C) ± 2	
		Melt-Quenching (m)	Microwave (w)	Melt-Quenching (m)	Microwave (w)	Melt-Quenching (m)	Microwave (w)
**PC**	PC5	2.477	2.476	283	280	383	382
	PC10	2.498	2.501	332	333	439	439
	PC15	2.525	2.528	388	387	493	492
	PC20	2.545	2.549	425	421	522	520
**PS**	PS1.5	2.541	2.540	445	446	579	578
	PS3	2.543	2.545	520	524	651	652
	PS4.5	2.546	2.548	536	534	655	654
	PS6	2.538	2.540	564	563	657	655

## Data Availability

The original contributions presented in this study are included in the article and Supplementary Materials. Further inquiries can be directed to the corresponding author.

## Supplementary Materials

The following supporting information can be downloaded at: https://www.mdpi.com/article/10.3390/ma18051079/s1, Figure S1: Macroscopic photograph of glass after aging. Figure S2: XRD pattern of glass after aging. Figure S3: FTIR spectrum of glass after aging. Figure S4: Relationship between total phosphorus, orthophosphate, polyphosphate concentrations over time in phosphate glass sample PC5 (a), PC10 (b), PC15 (c), PC20 (d), PS1.5 (e), PS3 (f), PS4.5 (g) and PS6 (h). Table S1: Comparison of scale inhibition efficiency of glass before and after aging. Table S2: Comparison between conventional and microwave melt−quenching methods.
