# Supplementary material for: Preparation of Phosphate Glass by the Conventional and Microwave Melt-Quenching Methods and Research on Its Performance
Source: Materials (Basel). 2025 Feb 28;18(5):1079. doi: 10.3390/ma18051079 (PMC11901276; doi:10.3390/ma18051079)
Supplement: Supplementary file 1 [file materials-18-01079-s001.zip › materials-3454684-supplementary.pdf]

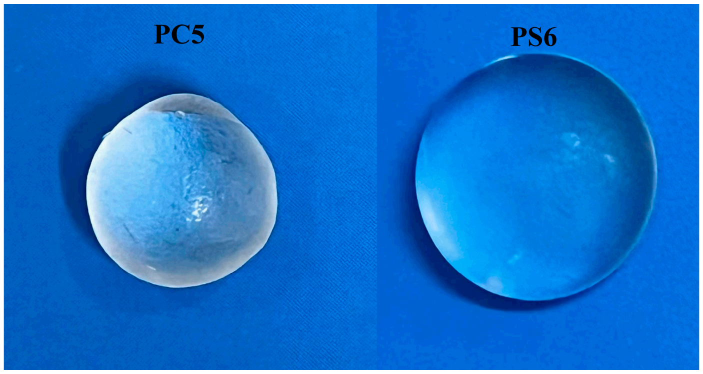

Figure S1. Macroscopic photograph of glass after aging.

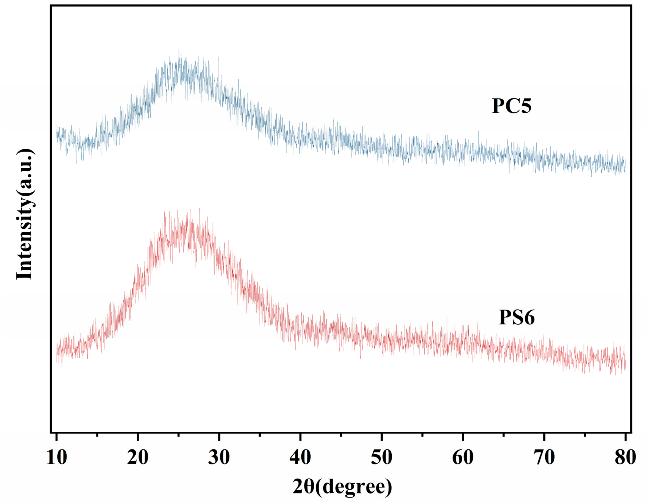

Figure S2. XRD pattern of glass after aging.

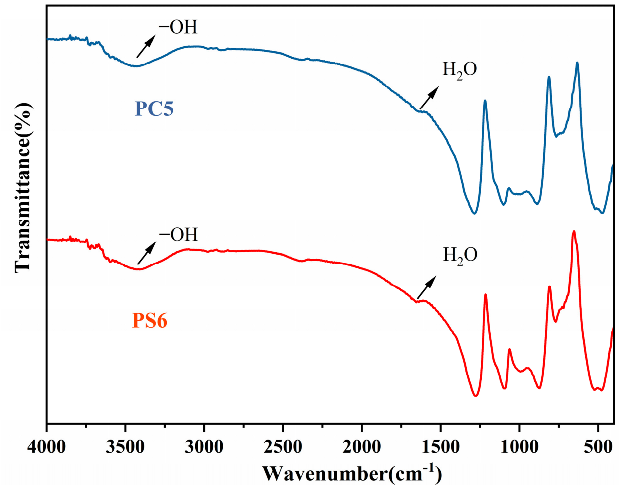

Figure S3. FTIR spectrum of glass after aging.

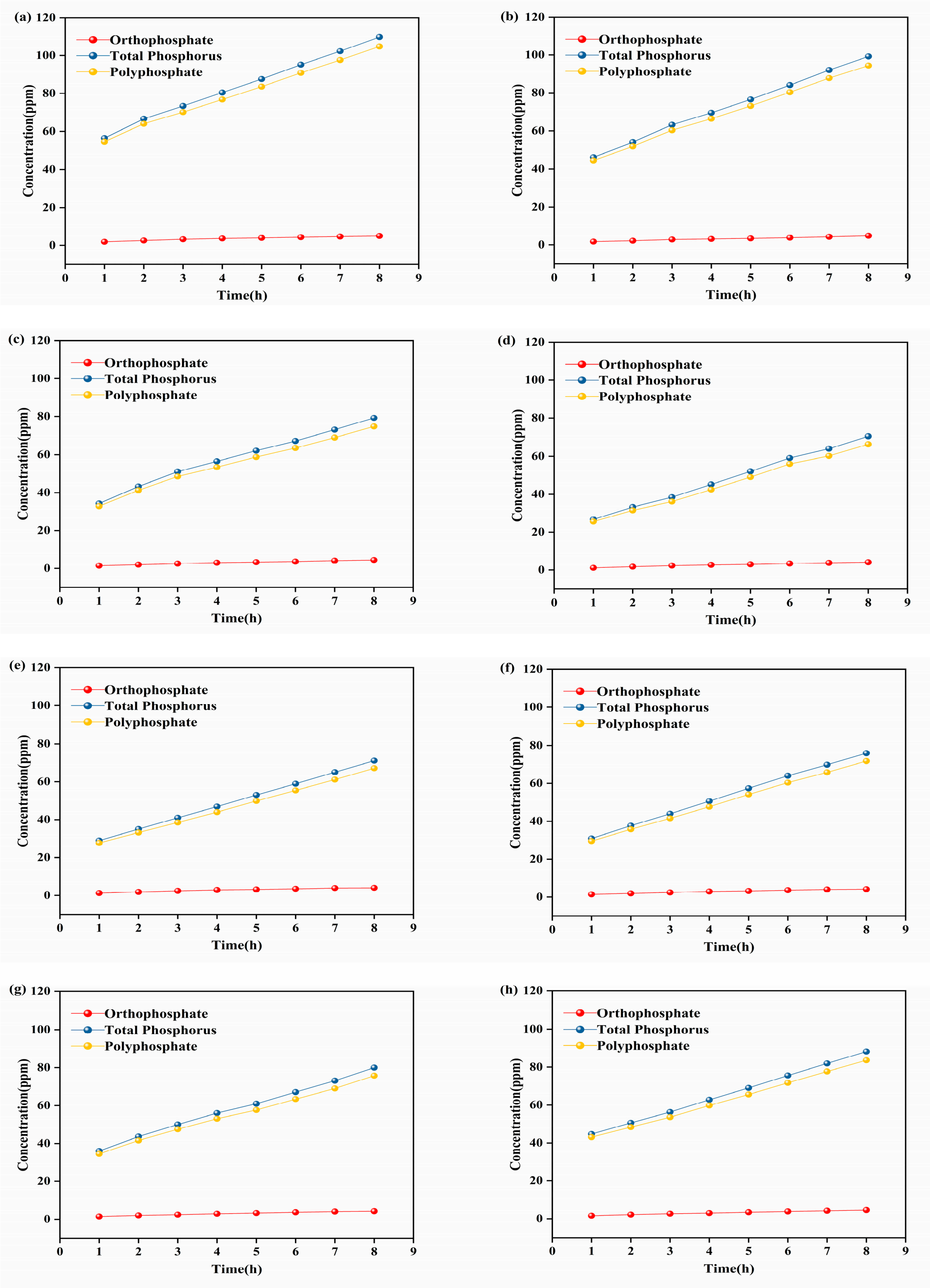

Figure S4. Relationship between total phosphorus, orthophosphate, polyphosphate concentrations over time in phosphate glass sample PC5 (a), PC10 (b), PC15 (c), PC20 (d), PS1.5 (e), PS3 (f), PS4.5 (g) and PS6 (h).

Table S1. Comparison of scale inhibition efficiency of glass before and after aging.

Sample Code	Scale Inhibition Efficiency(before aging)	Scale Inhibition Efficiency(after aging)
**PC5**	94.3%	93.2%
**PS6**	95.4%	93.8%

Table S2. Comparison between conventional and microwave melt−quenching methods.

Parameter	Conventional melt−quenching	Microwave melt−quenching
**XRD**	Amorphous and non−crystalline nature	Amorphous and non−crystalline nature
**FTIR and Raman**	The glass structure is mainly Q^2 unit, with a small amount of Q^1 unit	The glass structure is mainly Q^2 unit, with a small amount of Q^1 unit
**DTA**	The addition of CaO and SiO_2 will cause the increase of Tg(283→564℃) and Tc(383→657℃)	The addition of CaO and SiO_2 will cause the increase of Tg(280→563℃) and Tc(382→655℃)
**Density**	Except PC5 and PS1.5 have slightly lower densities	Except PC5 and PS1.5 have slightly higher densities
**Preparation time**	90min	20min
**Energy consumption**	High energy consumption	Low energy consumption
